# Sex difference in age and performance in elite Swiss freestyle swimmers competing from 50 m to 1,500 m

**DOI:** 10.1186/2193-1801-3-228

**Published:** 2014-05-06

**Authors:** Christoph Alexander Rüst, Thomas Rosemann, Beat Knechtle

**Affiliations:** Institute of General Practice and Health Services Research, University of Zurich, Zurich, Switzerland; Gesundheitszentrum St. Gallen, Vadianstrasse 26, 9001 St. Gallen, Switzerland

**Keywords:** Swimming, Aging, Endurance, Human

## Abstract

Recent studies reported different ages for peak freestyle swimming performances for 50 m and 1,500 m. The aims of the present study were (*i*) to determine the age of peak freestyle swimming speed for distances including 50 m, 100 m, 200 m, 400 m, 800 m, and 1,500 m and to (*ii*) analyze the sex difference in peak freestyle swimming speed for all distances between 50 m and 1,500 m for elite female and male swimmers competing at national level. Data from the ‘Swiss Swimming Federation’ between 2006 and 2010 from 10,405 men and 9,552 women were analyzed using regression analyses and analyses of variance (ANOVA). Women achieved peak freestyle swimming speed at ~20–21 years from 50 m to 400 m, at ~24–25 years in 1,500 m and at ~25–27 years in 800 m. In men, the age of peak freestyle swimming speed varied between ~22–23 years and ~25–27 years for 50 m to 1,500 m. Between the age of 10 and 29 years, the sex difference in freestyle swimming speed increased from 2.2 ± 0.4% to 19.0 ± 6.7% in 50 m (*r*^*2*^ = 0.87, *P* < 0.001), from 2.4 ± 0.7% to 10.8 ± 2.8% in 100 m (*r*^*2*^ = 0.67, *P* = 0.004) and from 3.6 ± 1.9% to 10.2 ± 3.4% in 200 m (*r*^*2*^ = 0.60, *P* = 0.008). In 400 m (*r*^*2*^ = 0.24), 800 m (*r*^*2*^ = 0.39) and 1,500 m (*r*^*2*^ = 0.34), the sex difference showed no changes (*P* > 0.05) with 6.9 ± 3.0%, 5.8 ± 3.5%, and 9.7 ± 8.6%, respectively. The sex difference in freestyle swimming speed showed no change with increasing race distance (*r*^*2*^ = 0.12, *P* > 0.05). To summarize, the age of peak freestyle swimming speed increased for women with the length of the race distance from 50 m to 200 m, but not from 400 m to 1,500 m. For men, the age of peak freestyle swimming speed varied between ~22–23 years and ~25–27 years from 50 m to 1,500 m. The sex difference in freestyle swimming speed of 9.1 ± 2.5% showed no change with increasing race distance. Future studies need to confirm these findings in elite swimmers competing at international level such as the World Championships and the Olympic Games.

## Background

Recent studies reported that the age-related decline in freestyle swimming performance depended on both the race distance and the sex of the athletes (Donato et al. 2003; Tanaka and Seals 1997). For example, it has been shown that, firstly, the age-related decline in freestyle swimming performance was greater for 1,500 m than for 50 m, and, secondly, this decline was more pronounced for women than for men in 50 m (Donato et al. 2003; Tanaka and Seals 1997). In women, the decline in swimming performance with advancing age from the 19- to 24-years to the 69- to 74-years age groups became progressively greater from the shortest distance (*i.e.* 50 m freestyle) to the two longest distances (*i.e.* 800 m and 1,500 m freestyle). For men, however, no difference in the magnitude of the performance decline with age between 100 m and 1,500 m freestyle was observed (Tanaka and Seals 1997).

For coaches and swimmers, the age of peak swimming speed in elite swimmers might be of greater interest than the age-related performance decline. Indeed, the knowledge of the age of peak swimming speed may help to plan the career of an athlete and support to reach the highest fitness level in a high-level competition such as the World Championships or the Olympic Games. The age of peak swimming speed has been investigated for freestyle swimmers for race distances ranging from 50 m to 1,500 m in pool swimming and also for ultra-distances in open-water swimming. For example, the age of peak freestyle swimming speed was found at ~21 years by Berthelot et al. ( 2012) when 12 events were chosen in long-course pool freestyle swimming (*i.e.* 50 m, 100 m, 200 m, 400 m, 800 m and 1,500 m for women and men, respectively). However, other authors reported different findings. Fairbrother ( 2007) demonstrated that the age of peak freestyle swimming speed in men’s 50 m was achieved in the late 20s and early 30s. Tanaka and Seals ( 1997) showed that peak freestyle swimming speed in 50 m was achieved at the age of ~20–30 years for both men and women. For ultra-swimmers, the fastest swimming speed was achieved in both women and men in the age group 30–39 years in a 12-hour in-door ultra-swimming event (Eichenberger et al. 2012). In open-water ultra-swimmers competing in the 46-km ‘Manhattan Island Marathon Swim’, the age of the annual three fastest swimmer increased from 28 ± 4 years (1983) to 38 ± 6 years (2013) in women and from 23 ± 4 years (1984) to 42 ± 8 years (2013) in men (Knechtle et al. 2014). In elite ultra-distance swimmers competing in the 5 km, 10 km and 25 km Fédération Internationale de Natation (FINA) World Cup swimming events, the age of peak swimming speed for the annual top ten women remained stable at 22.5 ± 1.2 years in 5 km, at 23.4 ± 0.9 years in 10 km and at 23.8 ± 0.9 years in 25 km. For the annual top ten men, the age of peak swimming speed increased from 23.7 ± 2.8 to 28.0 ± 5.1 years in 10 km but remained stable at 24.8 ± 1.0 years in 5 km and at 27.2 ± 1.1 years in 25 km (Zingg et al. 2014a).

Some studies suggested that a difference may exist in the age of peak freestyle swimming speed regarding both the race distance and the sex of the athletes (Schulz and Curnow 1988; Tanaka and Seals 1997; Zingg et al. 2014a). Schulz and Curnow ( 1988) reported that women generally reached their peak freestyle swimming speed at younger ages than men, especially on longer race distances such as the 800 m and the 1,500 m. These authors showed for freestyle swimming that for the 1896–1980 period, the age of peak freestyle swimming speed was ~21 years for 100 m, ~20 years for 400 m and ~20 years for 1,500 m for men, respectively (Schulz and Curnow 1988). For women, however, the peak freestyle swimming speed was attained at ~19 years for 100 m, at ~18 years for 400 m and at ~16 years for 800 m, respectively (Schulz and Curnow 1988). In contrast to these findings, Tanaka and Seals ( 1997) reported that men reached their fastest 1,500 m freestyle race times between 25 and 40 years whereas women achieved their best race times at the age of 30 to 35 years. For the shorter distances, peak freestyle swimming speed was attained at the age of 20 to 30 years for 50 m for both women and men (Tanaka and Seals 1997). In elite ultra-distance swimmers competing in the 5 km, 10 km and 25 km Fédération Internationale de Natation (FINA) World Cup swimming events, the age of peak performance was younger in women (~23 years) compared to men (~25–27 years) (Zingg et al. 2014a).

However, these reports for freestyle pool swimmers are based upon rather old data from 1896–1980 (Schulz and Curnow 1988), 1988–1999 (Donato et al. 2003), 1991–1995 (Tanaka and Seals 1997) and 1993–2001 (Fairbrother 2007). Moreover, these studies did not include the age group 10–19 years since Donato et al. ( 2003) investigated athletes aged from 19–85 years, Fairbrother ( 2007) from 19–96 years and Tanaka and Seals ( 1997) from 19–99 years. Regarding the sex difference in freestyle pool swimming, Tanaka and Seals ( 1997) reported that the sex differences were greatest for 50 m and 100 m freestyle and decreased with increasing race distance. The smallest sex difference was reported for the 1,500 m freestyle. Since Schulz and Curnow ( 1988) showed that the age of peak freestyle swimming speed in women was at ~20 years or below, the findings from the studies of Donato et al. ( 2003), Fairbrother ( 2007) and Tanaka and Seals ( 1997) need to be re-examined with the inclusion of the age group 10–19 years.

In this context, the aims of the present study were (*i*) to determine the age of peak freestyle swimming speed for distances including 50 m, 100 m, 200 m, 400 m, 800 m, and 1,500 m for elite female and male freestyle pool swimmers competing at national level (*i.e.* Switzerland) and to (*ii*) analyze the sex difference in peak freestyle swimming speed for all distances between 50 m and 1,500 m. According to present literature, it was hypothesized that (*i*) the age of peak freestyle swimming speed would be lower for women than for men for all distances from 50 m to 1,500 m and (*ii*) the sex difference in peak freestyle swimming speed would decrease with increasing race distance.

## Methods

### Ethics

The study was approved by the Institutional Review Board of St. Gallen, Switzerland, with waiver of the requirement for informed consent given that the study involved the analysis of publicly available data.

### Data sampling and data analysis

The data set for this study was obtained from the website of the ‘Swiss Swimming Federation’. The ‘Swiss Swimming Federation’ ( http://www.fsn.ch) keeps a high score list of the Swiss top swimmers ( http://rankings.fsn.ch) since 1984. Data from all freestyle swimmers from the Swiss swimming high score list between 1984 and 2010 were collected. Due to the low number of participants per age group in earlier years and missing data for women for the longer distances (*i.e.* 1,500 m) between 1984 and 2010, we restricted the data analysis to full data from five consecutive years between 2006 and 2010 in order to have a reliable data set. From this 5-year period, data from 19,957 athletes including 10,405 men and 9,552 women were available.

For the analysis of freestyle swimming speed of the different age groups, all athletes were separated by sex categorized in ten age groups <10 years, 10–19 years, 20–29 years, 30–39 years, 40–49 years, 50–59 years, 60–69 years, 70–79 years, 80–89 years, and >90 years. For each age group and sex, the annual three fastest race times of each distance (*i.e.* 50 m, 100 m, 200 m, 400 m, 800 m, and 1,500 m freestyle) were determined. Regression analysis for the top three athletes showed that neither age nor race times changed across this 5-year period. Since the Swiss high score list records only the fastest race time achieved by a swimmer per calendar year, no swimmer was included repeatedly for analysis. The top three results of each age group were pooled in order to get more reliable data (*n* = 15 for each age group). If there were less than three swimmers in an age group, the data were excluded from analysis.

Due to the low number of participants, swimmers in the age groups < 10 years and > 60 years had to be excluded from data analyses. The analyses showed that the fastest swimming speeds at all distances were achieved by athletes in the age groups 10–19 years or 20–29 years. We divided these two age groups into ten age groups of 2-year intervals such as 10–11 years, 12–13 years, 14–15 years, 16–17 years, 18–19 years, 20–21 years, 22–23 years, 24–25 years, 26–27, and 28–29 years. This corresponds to the official age groups for youth swimmers in Switzerland below the age of 20 years. For each age group, athletes with the top three swimming speeds per distance were determined, pooled and analyzed. Race time as a measure of swimming performance was converted to swimming speed (m/s) by calculating [race distance (m)]/[race time (s)]. The sex difference in swimming speed was calculated using the formula ([swimming speed in women] – [swimming speed in men])/[swimming speed in men] × 100. To calculate the sex difference as a function of distance, the mean sex difference of all 2-year intervals per distance were determined and analyzed (*n* = 30 for each distance).

### Statistical analysis

Each set of data was tested for normal distribution and for homogeneity of variances prior to statistical analyses. Normal distribution was tested using a D’Agostino and Pearson omnibus normality test and homogeneity of variances was tested using a Bartlett’s test. The changes in sex difference over age groups and over distance were determined using linear regression analyses. The differences in swimming speed and the sex difference between age groups within one distance were analyzed using one-way analysis of variance (ANOVA) with subsequent Tukey-Kramer post-hoc analysis. To find an interaction between age and race distance on sex difference, a two-way ANOVA (age group × distance) with subsequent Bonferroni post-hoc test was used. Statistical analyses were performed using IBM SPSS Statistics (Version 19, IBM SPSS, Chicago, IL, USA) and GraphPad Prism (Version 5, GraphPad Software, La Jolla, CA, USA). Significance was accepted at *P* < 0.05 (two-sided for *t*-tests).

## Results

**Figure 1:**
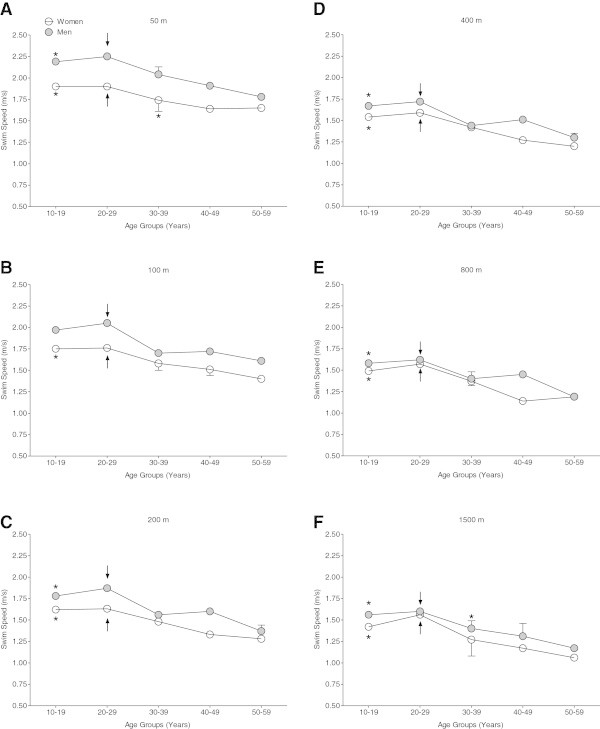
**Swimming speed of the top three athletes per age group for women and men.** 50 m freestyle **(Panel** **A** **)**, 100 m freestyle **(Panel** **B)**, 200 m freestyle **(Panel** **C)**, 400 m freestyle **(Panel** **D)**, 800 m freestyle **(Panel** **E** **)** and 1,500 m freestyle **(Panel** **F** **)**. For all distances, swimmers in the age group 20–29 years were the fastest for both women and men. The arrow (↓) indicates the fastest age group in women and men for all distances and an asterisk (*) indicates age groups that were not significantly different from the fastest age group (*P* < 0.05).

The fastest swimming speeds were achieved for all distances by both female and male athletes ranked in the age group 20–29 years regarding the swimming speed across the 10-year age groups (Figure 1). For both women and men, swimmers in the age group 10–19 years achieved a similar level of swimming speed compared to swimmers in the age group 20–29 years over all distances, except for men in 100 m freestyle. Additionally, women in 50 m freestyle and men in 1,500 m freestyle in the age group 30–39 years achieved a similar level of swimming speed compared to swimmers in the age group 20–29 years.

**Figure 2:**
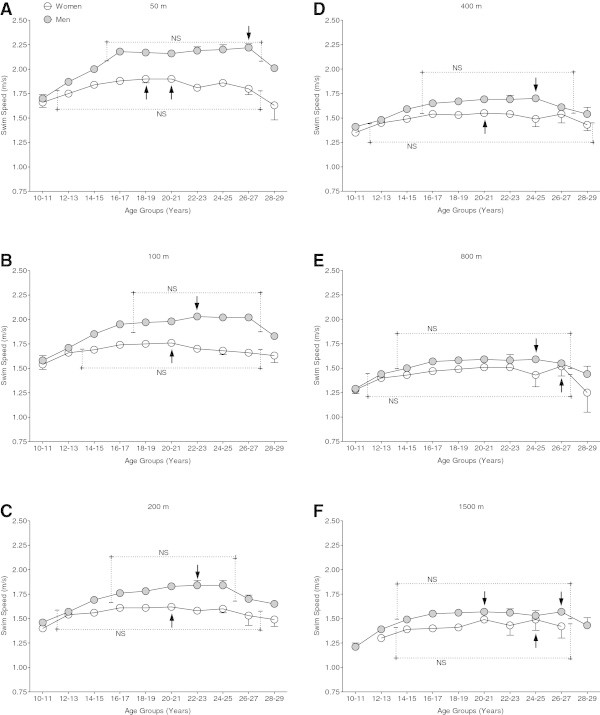
**Swimming speed of the top three athletes with 2-years age groups for athletes between 10 and 29 years for the different distances.** 50 m freestyle **(Panel** **A** **)**, 100 m freestyle **(Panel** **B** **)**, 200 m freestyle **(Panel** **C** **)**, 400 m freestyle **(Panel** **D** **)**, 800 m freestyle **(Panel** **E** **)** and 1,500 m freestyle **(Panel** **F** **)**. The arrow indicates the fastest age group and a rectangle indicated with ‘NS’ shows age groups not significantly different from the fastest age group.

The swimming speed in the two age groups 10–19 and 20–29 years was analyzed in details in 2-years intervals (Figure 2). Women achieved peak swimming speed at the age of ~20–21 years between 50 m and 400 m freestyle. The age of peak swimming speed increased to ~26–27 years for 800 m freestyle and to ~24–25 years for 1,500 m freestyle. In men, the age of peak swimming speed was between ~22–23 years and ~26–27 years from 50 m to 1,500 m freestyle. In 1,500 m freestyle, men aged ~20–21 years were able to achieve the same swimming speed than athletes at the age of ~25–27 years.

**Figure 3:**
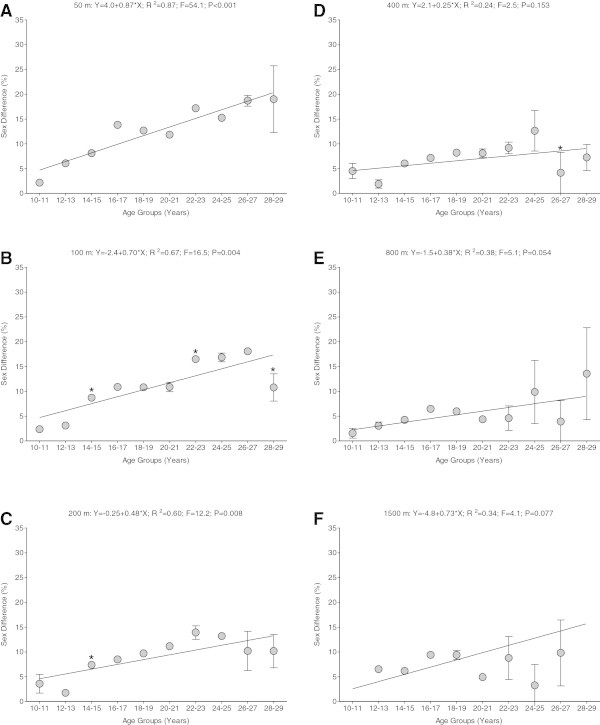
**The sex difference in swimming speed for the age groups of 2-year intervals from 10 to 29 years for the different distances.** 50 m freestyle **(Panel A)**, 100 m freestyle **(Panel B)**, 200 m freestyle **(Panel C)**, 400 m freestyle **(Panel D)**, 800 m freestyle **(Panel E)** and 1,500 m freestyle **(Panel F)**. * = not significantly different from the previous one.

No change in the sex difference in swimming speed across the 10-year interval age groups appeared for 50 m freestyle (Y = 17.0-0.13 × X; *r*^*2*^ = 0.40; F = 1.98; *P* > 0.05), 100 m freestyle (Y = 10.8 + 0.02 × X; *r*^*2*^ = 0.02; F = 0.07; *P* > 0.05), 200 m freestyle (Y = 10.4-0.01 × X; *r*^*2*^ < 0.01; F < 0.01; *P* > 0.05), 400 m freestyle (Y = 5.7 + 0.08 × X; *r*^*2*^ = 0.06; F = 0.20; *P* > 0.05), 800 m freestyle (Y = 4.5 + 0.07 × X; *r*^*2*^ = 0.01; F = 0.01; *P* > 0.05) and 1,500 m freestyle (Y = 5.48 + 0.069 × X; *r*^*2*^ = 0.20; F = 0.74; *P* > 0.05), where X = lower end of age-group range (*e.g.* ten for the age group 10–19 years). Figure 3 shows the sex difference for the same distances in groups of 2-year intervals from 10 to 29 years. For 50 m freestyle (Y = -4.0 + 0.87 × X; *r*^*2*^ = 0.87; F = 54.1; *P* < 0.001), 100 m freestyle (Y = -2.4 + 0.70 × X; *r*^*2*^ = 0.67; F = 16.5; *P* = 0.004) and 200 m freestyle (Y = -0.25 + 0.48 × X; *r*^*2*^ = 0.60; F = 12.2; *P* = 0.008), the sex difference in swimming speed increased with increasing age (*P* < 0.05) from 2.2 ± 0.4% to 19.0 ± 6.7% for 50 m freestyle, from 2.4 ± 0.7% to 10.8 ± 2.8% for 100 m freestyle, and from 3.6 ± 1.9% to 10.2 ± 3.4% for 200 m freestyle. For 400 m freestyle (Y = 2.1 + 0.25 × X; *r*^*2*^ = 0.24; F = 2.5; *P* = 0.153), 800 m freestyle (Y = -1.5 + 0.38 × X; *r*^*2*^ = 0.38; F = 5.1; *P* = 0.054) and 1,500 m freestyle (Y = -4.8 + 0.73 × X; *r*^*2*^ = 0.34; F = 4.1; *P* = 0.077), where X = lower end of age-group range (*e.g.* ten for the age group 10–11 years), the sex difference in swimming speed showed no changes across the age groups (*P* > 0.05) and remained unchanged at 6.9 ± 3.0% for 400 m freestyle, at 5.8 ± 3.5% for 800 m freestyle, and at 9.7 ± 8.6% for 1,500 m freestyle.

**Figure 4:**
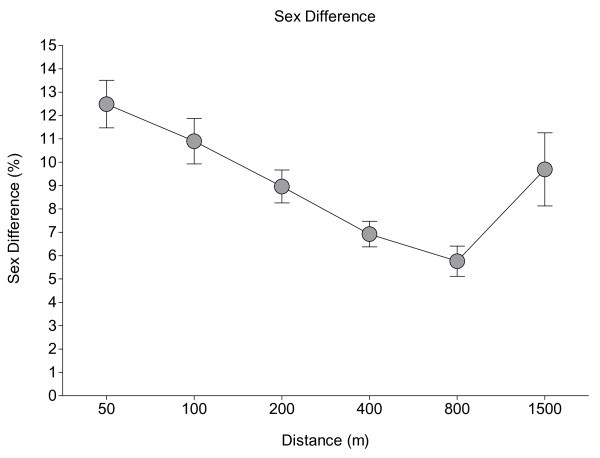
**The sex difference in swimming speed over the different distances.** For every distance, the top three swimmers of all 2-year age groups between 10 and 29 years were pooled, regardless of the year when they participated. Sex differences were not different across the distances.

There was a significant interaction between race distance and age on the sex difference in freestyle swimming speed. Race distance accounted for 14.9% of the total variance (F = 30.0; *P* < 0.001) and age for 37.2% of the total variance (F = 41.7; *P* < 0.001) of freestyle swimming speed. Interaction between age and race distance accounted for 36% (F = 8.0; *P* < 0.001) of the total variance. The relationship between sex difference and race distance showed no change in the sex difference with increasing length of the race distance (*r*^*2*^ = 0.12; *P* > 0.05) (Figure 4) and remained unchanged at 9.1 ± 2.5%.

## Discussion

This study intended to determine the age of peak swimming speed for both female and male elite freestyle pool swimmers for distances including 50 m, 100 m, 200 m, 400 m, 800 m, and 1,500 m, respectively, and to analyze the sex difference in the peak swimming speed for freestyle swimming from all distances between 50 m and 1,500 m. It was hypothesized that the age of peak freestyle swimming speed would be lower for women than for men for all distances and the sex difference in peak freestyle swimming speed would decrease with increasing race distance.

### The sex differences in the age of peak swimming speed

The first important finding was that the age of peak freestyle swimming speed was ~2 years higher in men than in women. In addition, the age of peak swimming speed increased for women with increasing race distance whereas it remained unchanged for men. We found a highly significant interaction between race distance and age on the sex difference where age accounted for 37.2% of the total variance of performance but race distance only 14.9%.

These findings differ from the results reported by Schulz and Curnow ( 1988) showing that younger ages were associated with increasing race distance for women when they analyzed Olympic gold medallists from 1896–1980. These disparate findings might be explained by the fact that we analyzed a 5-year time period from 2006–2010 and the age groups 10–19 years and 20–29 years in 2-years intervals whereas Schulz and Curnow ( 1988) separated their data into two halves from 1896–1936 and 1942–1980 using World War II as a dividing point. In Schulz and Curnow ( 1988), for men, the age of peak freestyle swimming speed decreased with increasing length of the race distance, *i.e.* 100 m freestyle (22.0 years and 20.78 years, respectively), 400 m freestyle (20.44 years and 19.44 years, respectively) and 1,500 m freestyle (21.09 years and 19.44 years, respectively). For women, however, the age of peak freestyle swimming speed increased with increasing length of the race distance, *i.e.* 100 m freestyle (18 years and 20.33 years, respectively) but decreased in 400 m freestyle (18 years and 17.33 years, respectively). For 800 m freestyle, there were no data available from 1896–1936 to compare with the second half after World War II.

While Schulz and Curnow ( 1988) investigated swimmers competing in a different time period (*i.e.* 1896–1980) compared to athletes in the present study (*i.e.* 2006–2010), anthropometric characteristics with an influence on swimming speed might have changed across years. Anthropometric characteristics such as body height (Avlonitou 1994; Geladas et al. 2005; Jagomägi and Jürimäe 2005; Zampagni et al. 2008) and body fat (Siders et al. 1993; Tuuri et al. 2002; Zuniga et al. 2011) have been found to be related to swimming performance. Changes in anthropometric characteristics across years such as body height may explain the differences in swimming performances between the sexes and the different time periods from the present subjects and the subjects in Schulz and Curnow ( 1988) since anthropometric characteristics of the swimmers examined during the 2006–2010 period were most probably different from those of swimmers investigated by Schulz and Curnow ( 1988) between 1896 and 1980. Also for elite athletes, body height, body mass and slenderness changed over time. Charles and Bejan ( 2009) reported that the improvement in the men’s 100 m freestyle world records between 1912 and 2008 were associated with an increase in body mass, body height and slenderness.

In the last century, both body height and body mass increased in humans. For example, between 1900 and 2000, Japanese boys had height and weight increments of 1.0–2.0 cm per decade and 0.4–1.7 kg per decade whereas girls had rates of 1.1–1.9 cm and 0.4–1.5 kg per decade (Kurokawa et al. 2008). The rates of height increment were significantly different between pre-, during and post-World War II periods. However, there seems to be a levelling-off in both body height and body weight across time. Kurokawa et al. ( 2008) reported the secular changes in growth status for children aged 11–12 years and 14–15 years since 1934. Between World War II and the early 1970s, both body height and body weight increased in both primary school children and junior high school students. During 1965–1974 and 1975–1984, an acceleration with a subsequent reduction in acceleration was observed for growth. During 1985–1994, a re-acceleration followed. From 1994 to 1999, body weight and body height increased in schoolchildren with a decrease between 1999 and 2003.

The different maturation between girls and boys during puberty could be a possible explanation for the sex difference in the age of peak freestyle swimming speed. The most important parameter in this context is body height (Charles and Bejan 2009). Wheeler ( 1991) described that peak growth velocity was not reached until the age of 12 years and 14 years in girls and boys, respectively. The onset of puberty expressed as the skeletal/biological age is at the age of ~11 years and ~13 years in girls and boys, respectively (Tanner et al. 1975). In a recent study from Martin et al. ( 2011), the sex difference from ~2 years found in other studies was confirmed in accordance with a different bone growth (*i.e.* metacarpal thickness, width, length and medullary diameter) between girls and boys.

Another explanation regarding anthropometric characteristics apart from body height for the different ages of peak swimming speed between women and men could be body fat as an important predictor variable for swimming performance (Siders et al. 1993; Tuuri et al. 2002; Zuniga et al. 2011). Siders et al. ( 1993) reported that body composition characteristics such as body fat and body mass may be important predictors for swimming performance in women but not in men and Tuuri et al. ( 2002) showed that a greater fat mass in female swimmers was more strongly related to lower levels of swimming performance.

The earlier onset of puberty in women might explain the age differences between the sexes. Bitar et al. ( 2000) reported an increase in fat-free mass during the onset of puberty for both boys and girls where the gain in fat mass was more pronounced in girls than in boys. Lean body mass reflecting skeletal muscle mass starts to increase during the early phase of puberty in both girls and boys. In girls, fat mass increases during the late phase of puberty in girls (Wheeler 1991). A further increase in fat mass after puberty may impair swimming performance in women. Zuniga et al. ( 2011) showed that boy (9.40% body fat) and girl (12.73% body fat) sprint swimmers at the age of ~11 years were different regarding percent body fat and suggested that swimming performance for girls may be improved through training programs designed to reduce body fatness.

This study found a difference of ~2 years in the age of peak freestyle swimming performance. Recent studies investigated also the age of peak swimming speed for other strokes such as backstroke (Kollarz et al. 2013a, 2013b), breaststroke (Wolfrum et al. 2013), butterfly (Zingg et al. 2014b) and individual medley (Buhl et al. 2013; Vaso et al. 2013). Generally, women achieved peak swimming speed at younger ages than men. For backstroke, Kollarz et al. ( 2013a, 2013b) described a sex difference in the age of peak swimming speed in swimmers competing at national level (*i.e.* Swiss elite swimmers) of ~2 years in 50 m and ~4 years in both 100 m and 200 m with women competing at younger ages than men. For breaststroke, Wolfrum et al. ( 2013) compared swimmers competing at national level ranked in the Swiss high score list during 2006–2010 and international swimmers competing in the finals of the FINA World Swimming Championships during 2003–2011. The age of peak swimming speed was ~2 years younger in breaststroke than in freestyle for women competing at national level (~18.5 years *versus* ~20.5 years, respectively) and for women competing at international level (~22.5 years *versus* ~24.5 years, respectively). Men competing at national level achieved peak swimming speed ~5 years earlier in breaststroke than in freestyle (~18.5 years *versus* ~23.8 years, respectively). The difference for men competing at international level was ~2 years (~24.5 years for breaststroke *versus* ~26.5 years for freestyle). The age of peak swimming speed was ~4 years older on average for women competing at international level than for women competing at national level irrespective of the discipline. Peak swimming speed was achieved ~6 years later in breaststroke and ~3 years later in freestyle for men competing at international level compared to men competing at national level. In butterfly, Zingg et al. ( 2014b) investigated swimmers competing at national level in 50 m, 100 m and 200 m between 1994 and 2011 where the fastest women were younger than the fastest men. For women, the age of peak swimming speed was ~19 years. For men, the age of peak butterfly swimming speed was at ~22 years over all distances. The age of peak butterfly swimming speed increased for women in 50 m from ~19 years to ~21 years, in 100 m and 200 m from ~18 years to ~20 years. For men, the age of peak butterfly swimming speed was unchanged at ~24 years, ~23 years and ~21 years, respectively. However, for individual medley in contrast to the other disciplines, the age of peak swimming speed was higher in men compared to women in athletes competing at national level with a sex difference of ~1–5 years with a mean age of peak swimming speed at ~20–21 years for women and ~22–25 years for men (Vaso et al. 2013). Buhl et al. ( 2013) analyzed potential changes in the age of peak individual medley swimming speed for both elite female and male Swiss swimmers from 1994 to 2011. The age of peak swimming speed was higher for men than for women in 200 m medley (~21.1 years *versus* ~18.2 years) and in 400 m medley (~20.8 years *versus* ~18.6 years). Obviously, the age of peak swimming speed is lower in women compared to men for freestyle, backstroke, breaststroke and butterfly, but not for individual medley where the opposite was found.

It seems that the age of peak swimming speed increased with increasing race distance in pool swimmers competing in freestyle (Berthelot et al. 2012; Schulz and Curnow 1988; Wolfrum et al. 2013), backstroke (Kollarz et al. 2013a), and individual medley (Buhl et al. 2013). However, the opposite was found in runners where younger athletes were faster in the shorter running distances (Krzysztof and Mero 2013; Schulz and Curnow 1988) and older athletes were faster in both marathons (Leyk et al. 2009; Trappe 2007) and ultra-marathons (Hoffman 2010; Knechtle et al. 2012). For sprint and middle-distance runners, the age of peak running speed was ~20 years for women and ~23 years for men in 100 m and ~24.5 years for women and ~24 years for men in 800 m. Krzysztof and Mero ( 2013) described the age of peak running performance of male 100 m sprinters at ~25–27 years. For longer running distances such as marathon and ultra-marathon distances, the age of peak marathon performance was ~20–55 years (Leyk et al. 2009) and ~25–35 years (Trappe 2007) for both women and men. For elite marathoners competing in the seven marathons of the World Marathon Majors Series, women (~29.8 years) were older than men (~28.9 years), but for only two of the seven marathons, the Chicago and the London marathons where the sex difference in age was not consistent across the years (Hunter et al. 2011). There was no sex difference in age for the Berlin, Boston, New York City, World Championship, and Olympic marathons (Hunter et al. 2011).

For ultra-marathoners, age and running speed of the annual fastest women and men in all 100 km ultra-marathons held worldwide between 1960 and 2012 were analyzed in 148,017 finishes with 18,998 women and 129,019 men (Cejka et al. 2014). The fastest race times in 100 km were achieved at ~34.9 years for women and at ~34.5 years in men (Cejka et al. 2014). For ultra-marathoners competing in 161 km ultra-marathons, race times and ages of the annual ten fastest women and men were analyzed for 35,956 finishes (6,862 for women and 29,094 for men) competing between 1998 and 2011 (Rüst et al. 2013). The mean ages of the annual top ten runners were ~39.2 years for women and ~37.2 years for men. The age of peak running performance was not different between women and men and showed no changes across the years (Rüst et al. 2013).

### The sex differences in peak freestyle swimming speed

A second important finding was the sex difference in peak freestyle swimming speed did not significantly change with increasing race distance. Our findings for the distances from 100 m to 400 m freestyle are in line with previous findings from Nevill et al. ( 2007). Their analysis of the 100 m, 200 m, and 400 m freestyle swimming world records from 1957 to 2006 showed that the sex difference in swimming performance remained unchanged between ~8% and ~11%. Regarding the distance, the sex difference decreased with increasing race distance. For 100 m freestyle, the sex difference was at ~11%, for 200 m freestyle at ~10% and for 400 m freestyle at ~9%, respectively (Nevill et al. 2007).

For the longer distances of 800 m and 1,500 m freestyle, however, our findings do not agree with the findings from Tanaka and Seals ( 1997). These authors reported that the sex differences were greater for 50 m and 100 m freestyle and decreased with increasing race distance. For 1,500 m freestyle, the lowest sex difference was observed. They showed a sex difference of ~20% for 50 m freestyle with a continuous decrease in sex difference to ~10% for 1,500 m freestyle. They found significant differences between any paired swimming distances except for the 50 m *versus* 100 m freestyle and 800 m *versus* 1,500 m freestyle.

The most probable explanation for these different findings was the inclusion of the age group 10–19 years in the present investigation compared to the sample in Tanaka and Seals ( 1997) without inclusion of this age group. While Tanaka and Seals ( 1997) investigated participants aged from 19–99 years competing in the US Masters Swimming Championships from 1991–1995, we investigated elite Swiss swimmers aged from 10–59 years and competing between 2006 and 2010. Because the fastest race times were achieved for most distances in athletes aged 10 and 29 years, the inclusion of the 10–19 years age group in the present study may explain the difference in swimming speed in the sex difference for the longer race distances. There is a physiological sex difference explaining the faster performance of men compared to women. Generally, men have larger hearts, a lower body fat, a larger haemoglobin concentration and a higher skeletal muscle mass per unit of body weight, and a higher maximal oxygen consumption than women (Cheuvront et al. 2005; Joyner 1993; Sparling 1980).

### Limitations of the present study and implications for future research

This study is limited that several variables were not included. We did not know whether these athletes were competing at a recreational or a professional level (*i.e.* if one swims professionally and exclusively, or at the same time studying or working, for example), the start and the end of a swimming career (*i.e.* if only a few swimmers compete professionally past a certain age then the sample size might be skewed towards certain ages), technological advances in swimming suits and between-swimmer differences in the swimming suits used (*e.g.* full-body, half body etc.), current (and history of) scientific support of each athlete (*e.g.* support provided by biomechanists, physiologists, sport psychologists, nutritionists, etc.), personal and socio-economic factors, long and short-term plans, considered together with injuries and illnesses data (*e.g.* what competitions swimmers might be focusing on over a period of years, and how any injuries or illnesses might have affected the participation or performance in these competitions). These missing aspects may be included in future studies. The present findings for swimmers competing at national level should be confirmed in elite swimmers competing at international level such as the World Championships or the Olympic Games.

## Conclusions

For elite freestyle swimmers competing at national level, the age of peak freestyle swimming speed was ~2 years older in men compared to women and increased with increasing length of the race distance for women, but not for men. Female swimmers achieved peak freestyle swimming speed at ~20–21 years for distances comprised between 50 m and 400 m freestyle. The age of peak freestyle swimming speed increased to ~24–25 years for 1,500 m freestyle and to ~25–27 years for 800 m freestyle, respectively. In men, the age of peak freestyle swimming speed was between ~22–23 years and ~25–27 years for distances comprised between 50 m and 1,500 m freestyle, respectively. For coaches and athletes, women seem to achieve their fastest swimming speeds for shorter distances at lower ages (~20–21 years) compared to longer distances (~25–27 years). Men, however, seem to achieve the fastest freestyle swimming speed between 22 and 27 years. Furthermore, the sex difference in peak freestyle swimming speed did not significantly change with increasing race distance.
